# The Physical Activity Environment Policy Index for monitoring government policies and actions to improve physical activity

**DOI:** 10.1093/eurpub/ckac062

**Published:** 2022-11-29

**Authors:** Catherine B Woods, Liam Kelly, Kevin Volf, Peter Gelius, Sven Messing, Sarah Forberger, Jeroen Lakerveld, Nicolette R den Braver, Joanna Zukowska, Enrique García Bengoechea

**Affiliations:** Department of Physical Education and Sport Sciences, Physical Activity for Health Research Cluster, Health Research Institute, University of Limerick, Limerick, Ireland; Department of Physical Education and Sport Sciences, Physical Activity for Health Research Cluster, Health Research Institute, University of Limerick, Limerick, Ireland; Department of Physical Education and Sport Sciences, Physical Activity for Health Research Cluster, Health Research Institute, University of Limerick, Limerick, Ireland; Department of Sport Science and Sport, Friedrich-Alexander-Universität Erlangen-Nürnberg, Erlangen, Germany; Department of Sport Science and Sport, Friedrich-Alexander-Universität Erlangen-Nürnberg, Erlangen, Germany; Leibniz Institute for Prevention Research and Epidemiology—BIPS, Bremen, Germany; Department of Epidemiology and Data Science, Amsterdam UMC, VU University Amsterdam, Amsterdam Public Health Research institute, Amsterdam, The Netherlands; Upstream Team, Amsterdam UMC, VU University Amsterdam, Amsterdam, The Netherlands; Department of Epidemiology and Data Science, Amsterdam UMC, VU University Amsterdam, Amsterdam Public Health Research institute, Amsterdam, The Netherlands; Upstream Team, Amsterdam UMC, VU University Amsterdam, Amsterdam, The Netherlands; Faculty of Civil and Environmental Engineering, Gdansk University of Technology, Gdansk, Poland; Department of Physical Education and Sport Sciences, Physical Activity for Health Research Cluster, Health Research Institute, University of Limerick, Limerick, Ireland; Research and Innovation Unit, Sport Ireland, Dublin, Ireland

## Abstract

**Background:**

A multifaceted response, including government action, is essential to improve population levels of physical activity (PA). This article describes the development process of the ‘Physical Activity Environment Policy Index’ (PA-EPI) monitoring framework, a tool to assess government policies and actions for creating a healthy PA environment.

**Methods:**

An iterative process was undertaken. This involved a review of policy documents from authoritative organizations, a PA policy audit of four European countries, and a systematic review of scientific literature. This was followed by an online consultation with academic experts (*N* = 101; 20 countries, 72% response rate), and policymakers (*N* = 40, 4 EU countries). During this process, consensus workshops were conducted, where quantitative and qualitative data, alongside theoretical and pragmatic considerations, were used to inform PA-EPI development.

**Results:**

The PA-EPI is conceptualized as a two-component ‘policy’ and ‘infrastructure support’ framework. The two-components comprise eight policy and seven infrastructure support domains. The policy domains are education, transport, urban design, healthcare, public education (including mass media), sport-for-all, workplaces and community. The infrastructure support domains are leadership, governance, monitoring and intelligence, funding and resources, platforms for interaction, workforce development and health-in-all-policies. Forty-five ‘good practice statements’ or indicators of ideal good practice within each domain conclude the PA-EPI. A potential eight-step process for conducting the PA-EPI is described.

**Conclusions:**

Once pre-tested and piloted in several countries of various sizes and income levels, the PA-EPI good practice statements will evolve into benchmarks established by governments at the forefront of creating and implementing policies to address inactivity.

## Introduction

Worldwide, 71% of all deaths are attributed to non-communicable diseases (NCDs),^1^ with the combined burden of physical inactivity, poor (quality) diet and high body mass index accounting for 11.9% of disability adjusted life years in 2019.^2^ The World Health Organization’s (WHO) Global Action Plan for the prevention and control of NCDs 2013–20 set a target of a reduction of NCD-related mortality by 25% by the year 2025 and identified increasing population levels of physical activity as necessary to achieve this goal. The importance of addressing physical inactivity as a public health priority has grown, as evidenced in the establishment of the Active Healthy Kids Global Alliance,^3^ the Global Observatory for Physical Activity (GoPA!)^4^; and the European Union/WHO PA factsheets to monitor the state of PA surveillance, research and policy worldwide.^5^^,^^6^ The 74th World Health Assembly in April 2021, a midpoint evaluation of the WHO NCD action plan, documented good progress at a country level regarding the introduction of national policies for PA, but minimal progress on addressing population levels of physical inactivity.^7^ Indeed, trend data over the last decade show no reduction, at a national and global level, of the high proportion of the population that remains inactive or do not meet WHO PA guidelines, of at least 150 min moderate-to-vigorous PA per week for adults.^8^ The WHO Global Action Plan on Physical Activity 2018–30 has set a target of a 15% relative reduction in the prevalence of population inactivity by 2030, and linked the promotion of PA to the UN’s Sustainable Development Goals.^9^

A substantial body of literature exists on solutions that can potentially address this inactivity challenge. An ecological and multi-level,^10^^,^^11^ as well as a comprehensive whole system approach^9^ has been recommended. These approaches have been used previously to successfully reduce the use of tobacco products^12^ and develop food environments supportive of healthy dietary behaviours.^13^ To address physical inactivity, a ‘healthy’ PA environment is paramount. The PA environment is defined, for the purpose of this article, as the collective physical, economic, political and sociocultural contexts, opportunities and conditions that influence one’s PA choices and behaviours. An unhealthy PA environment may be caused by a lack of ‘upstream’ policy progress in domains known to have a positive impact on PA behaviour, and when combined with a lack of effective infrastructure support for policy implementation,^13^ then the inactivity pandemic^14^ is likely to sustain, as the ‘system’ or environment remains unchanged despite best ‘downstream’ or programmatic efforts. To better understand the PA policy environment, we selected eight policy domains, representing multiple sectors, based on the International Society for Physical Activity and Health’s (ISPAH) Eight Investments that work for PA.^15^ ISPAH provides good evidence of effectiveness for each of these domains, and a rationale for investment in these areas due to their worldwide applicability and their potential to tackle inactivity if addressed comprehensively through a systems approach. These domains are transport, urban design, education, healthcare, community-wide programmes, sport and recreation for all, workplaces and public education. Public policy interventions can help with the creation of a supportive environment for PA.^13^ Government policy action can use a systems approach to leverage and integrate multiple sectors—such as those listed above—in partnership to create a healthy PA environment with sustainable effects. However, there is a knowledge gap regarding how to independently assess government progress in implementing public policies within these domains to create this healthy PA environment.

The public sector refers to all levels of government, from international to local, and while we mainly refer here to national governments, our findings can also be applied to sub-national levels, where relevant. Policies are defined as ‘decisions, plans, and actions that are enforced by national or regional governments or their agencies (including at the local level), which may directly or indirectly achieve specific (health) goals within a society’.^16^ While policy research in the field of physical activity and public health is relatively recent, there has been a rapid growth in research describing national policy approaches to PA. This has shown that many countries worldwide have formal written PA policies, guidelines, monitoring systems and national PA targets.^17^ A recent survey of officials representing national ministries in the EU Physical Activity Focal Point Network indicated that governments appreciate regular, systematic and comparative monitoring of their national PA policies.^18^ According to the focal points, results from monitoring systems are useful to take stock of the situation in their own countries, to gain insights into developments in other countries, to foster communication between sectors and to develop for national policy. However, knowledge of the status, implementation and effectiveness of policies that can promote PA at a country level is still limited, with no clear guidance on which policies governments should preferably use in different settings or under various preconditions.^19^ In addition, research on the role of sub-national or local government in the implementation of these national policies, or indeed in locally developed policies to promote PA, is inadequate.^20^ Research is needed on what constitutes best practice public sector policies to promote PA, and on how to evaluate their impacts and their level of implementation.^21^

The Comprehensive Analysis of Policy on Physical Activity (CAPPA) was developed to guide research related to PA policy analysis. It consists of six categories including purpose of the analysis, policy level, policy sector, type of policy, stage of policy cycle and scope of the analysis.^21^ A follow-up systematic review identified several instruments for the purpose of PA policy auditing (the documentation of the presence or absence of policies and their component parts), and for assessment of national-level PA policies.^22^ However, the authors noted that none of the instruments covered all six components of the CAPPA framework.^22^ Additionally, no reference was made to instruments that would facilitate evaluation of implementation of public sector policies related to PA, independent of government.

The ‘Policy Evaluation Network’ (PEN) is a multi-disciplinary research network established for the monitoring, benchmarking and evaluation of policies that affect diet, PA and sedentary behaviour with a standardized approach across Europe.^16^ This manuscript is based on learnings from the INFORMAS Food-EPI,^13^ adapted to answer the question ‘How much progress have governments made towards good practice in improving the PA environment and implementing physical inactivity/NCD prevention policies and actions?’ This involved the development of the first ‘Physical Activity Environment Policy Index’ (PA-EPI), which this article describes. The PA-EPI is a tool that can be used to monitor and benchmark public sector PA policies and actions, the latter involving a panel of experts independent of government policy makers. This article also outlines the projected steps in the use of the PA-EPI to compare government policies, over time and across countries, to stimulate actions to improve the healthiness of the physical activity environment. These steps are modelled after the INFORMAS monitoring framework^23^ currently used in 30 countries worldwide (see: https://www.informas.org/countries/).

## Development of a PA-EPI framework for monitoring government policies and actions

Overall, the development of the PA-EPI involved an iterative process consisting of four steps. Step 1 involved a desk-based research on key policy documents, PA policy audits and a scientific literature review, to generate the PA-EPI framework and create a draft PA-EPI monitoring tool. An online consultation process was organized for step three involving academic experts and policymaker experts to revise and refine, as appropriate, the PA-EPI monitoring tool. Throughout the process, PEN consensus workshops were undertaken to synthesize the information generated (steps two and four). The four steps are further detailed below:

### Step 1: policy document review, policy audit and scientific literature synthesis

Three methodologies were used, (i) policy document review, (ii) policy audit and (iii) a review of the scientific literature. Using tacit knowledge of the research team and in consultation with PEN experts, we identified and reviewed policy documents, such as authoritative evidence-based reports or expert committee documents on the promotion of PA from international and supranational organizations (e.g. WHO, UNESCO, World Health Assembly and Council of Europe), national government agencies (e.g. Departments of Health, Sport, Transport etc.), global non-government organizations (e.g. World Cancer Research Foundation) and professional societies (e.g. International Society of Physical Activity and Health) for their recommendations in relation to improving the PA environment and PA behaviour (see Supplementary table SA for documents). In addition, to understand the status of PA policy development more fully, we audited national PA policies in four European countries using the HEPA PAT, a tool provided by WHO that facilitates a country-specific situational analysis and international comparison.^19^ This provided us with a detailed knowledge of governments’ policy-making structures and how they engage in agenda-setting, policy formulation, decision-making, policy implementation and policy evaluation. It also allowed us to identify key PA policymakers for the online consultation phase of the PA-EPI development. In parallel, to assess the level and quality of peer-reviewed support for the impact of public policies on PA, we consolidated the evidence from existing literature reviews, including from the DEDIPAC systematic reviews on determinants of PA e.g.^24^ and where gaps were found, we undertook new reviews of empirical studies^25–28^ and an umbrella review.^29^ PEN researchers mapped, reviewed and synthesized all policy actions and recommendations from these three methodologies during an extensive inductive and deductive process. This led to the development of the PA-EPI framework.

### Step 2: PEN consensus on the PA-EPI framework prototype

**Figure 1:**
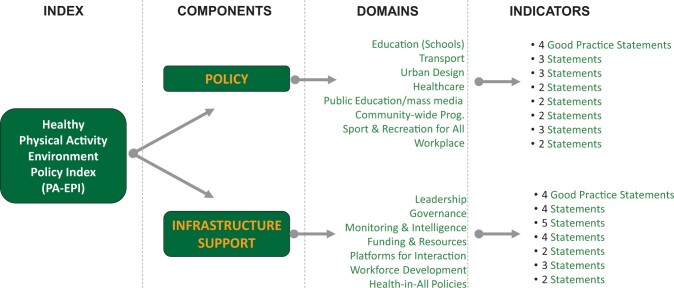
The PA-EPI framework (including the final number of good practice statements for each domain (*N* = 45))

The proposed PA-EPI framework was conceptualized as including two-components ‘policy’ and ‘infrastructure support’. Within the framework’s two components, eight policy and seven infrastructure support domains were created (figure 1). The policy domains align with ISPAH’s ‘Eight investments that work for physical activity’,^15^ and are education, transport, urban design, healthcare, public education (including mass media), sport-for-all, workplaces and community. The infrastructure support domains align with those of the INFORMAS Food-EPI,^13^ and are leadership, governance, monitoring and intelligence, funding and resources, platforms for interaction, workforce development and health-in-all-policies. The latter were based on the existing WHO system-building blocks,^30^ with an additional ‘health in- all-policies’ (or ‘policy alignment’) domain added to emphasize the importance of considering health in the development of non-health policies.^31^ Each of the 15 PA-EPI domains contains an ‘ideal good practice’, as well as examples of ‘good practice statements’ (or indicators of this ideal good practice). These statements were formulated following consensus workshops by the PEN researchers, based on the specific recommendations derived from the three methodological processes described above, and using an iterative process. Thus, 15 examples of ideal good practice, comprised of 53 good practice statements (30 for the policy domain and 23 for infrastructure support) were included in the prototype PA-EPI framework.

### Step 3: academic and policymaker expert consultation

A strength of the formulation of the PA-EPI good practice statements was their foundation in policy documents, policy practice and/or scientific evidence. However, for added rigor and credibility, a further expert consultation with individuals beyond the PEN network was deemed necessary. Ethical approval was obtained from the Research Ethics Committee of the Faculty of Education and Health Sciences, University of Limerick (2021_03_04_EHS), and with participant consent, we conducted an online consultation process with academic and policymaker experts. Academic experts were identified through the PEN network and authorship of publications identified in Step 1. Inclusion criteria were substantial track record (number of years of experience and/or number of peer-reviewed publications) in PA policy research and/or in policy domain specific policy research. Policymaker experts were recruited from the four PEN countries who completed the HEPA PAT^19^ in Step 1 (Ireland, Germany, The Netherlands and Poland). A quota sampling technique^32^ was used with the aim of having at least one policymaker per PA-EPI policy domain, ideally per country. Recruitment involved emailing each expert to invite them to participate in the consultation process.

The online consultation had two aims. Aim 1 was to gather opinion and advice regarding the formulation and the evidence base for the ideal good practice and the good practice statements, consequently only academic experts were invited. Every identified expert was invited to complete an online PA-EPI survey, this involved reading each statement and recommending its inclusion or removal from the PA-EPI tool. If the statement was recommended for inclusion, experts could select ‘should be totally changed’, ‘kept with some adaptation’ or ‘kept without change’. For each response, experts provided a rationale and suggestions on alternative wording, where relevant. Academic experts (*N* = 101) from 20 countries were invited; 72% (*n* = 73) replied, and of this number, 71% (*n* = 52) completed, 19% (*n* = 14) partially completed and 10% (*n* = 7) declined to complete the survey. Experts provided 885 qualitative comments and this data were used to improve the wording of the good practice statements. Feedback from experts also requested greater clarity on terminology, on the intended/desired PA-EPI policy level to be addressed (national or sub-national), and on the implementation/evaluation aspect of the PA-EPI. Full analysis of expert responses determined a decrease in the PA-EPI policy domain good practice statements from 30 to 26, while the infrastructure domain statements increased from 23 to 26 statements. This revised tool formed the basis for the next phase, of the online consultation.

The second aim of the online consultation was to reduce the number of good practice statements further based on both academic and policymaker evaluation of each statement for its importance, feasibility and ease of assessment for improving the PA environment and/or PA behaviour. A 10-point Likert scale was used to evaluate each statement on (i) its importance for increasing population levels of PA (relatively unimportant to extremely important), (ii) how feasible (practical, achievable) it is for governments to implement this good practice statement (relatively unfeasible to extremely feasible) and (iii) how easy it is for governments to assess the extent of implementation of the good practice statement (not at all easy to assess to very easy to assess). Experts could also provide feedback on the tool overall. All 66 academic experts who consented to Stage 1 of the online consultation were invited to Stage 2, and 75% (*n* = 50) participated. For policymakers, our aim was to have at least one national policymaker per policy domain per country. More PA policy experts in the traditional health (*n* = 16), sport (*n* = 7) and education (*n* = 5) policy domains, in comparison to the less traditional areas of transport (*n* = 2), urban design (*n* = 2), community (*n* = 4), public education (*n* = 3) or workplace (*n* = 0) were recruited. Overall, with 40 policymakers contributing, we were confident in the policymaker reviews of the good practice statements based on the consultation criteria. Table 1 shows the policy and the infrastructure support domain median scores by group across the criteria of importance, feasibility and ease of assessment. Data analysis placed 21 good practice statements below the median in all three criteria, importance, feasibility and ease of assessment, for both groups (Supplementary table SB), and were therefore removed from further analysis.

**Table 1:** Domain good practice statement (median) scores following Academic and Policymaker Expert Consultation

Good practice statement	Academic				Policymakers			
	*I*	*F*	*A*	Median	*I*	*F*	*A*	Median
Policy domains								
Education	9.1	7.3	7.3	8.0	8.6	7.0	6.6	7.3
Transport	9.0	7.0	7.4	7.9	8.4	7.0	6.5	7.2
Urban design	9.0	6.4	6.8	7.4	8.6	6.0	5.9	6.8
Sport & Recreation for All	8.1	6.8	6.8	7.2	8.3	7.2	6.6	7.4
Healthcare	8.2	6.4	6.6	7.1	8.0	6.6	6.0	6.8
Public education/mass media	7.5	6.8	6.5	6.9	7.5	6.8	6.3	6.9
Workplace	8.1	6.3	6.4	6.9	8.1	6.7	6.0	6.8
Community	7.9	6.4	6.0	6.8	8.3	7.1	6.7	7.3
Policy domain median	**8.2**	**6.6**	**6.7**	**7.2**	**8.3**	**6.9**	**6.4**	**7.0**
Infrastructure support domains								
Leadership	8.9	7.0	7.2	7.8	8.4	7.0	6.7	7.4
Monitoring and intelligence	9.2	7.0	7.0	7.7	8.3	6.6	6.5	7.2
Workforce development	8.5	6.9	6.9	7.4	7.9	6.2	6.2	6.8
Funding and resources	8.8	6.1	7.1	7.3	8.1	6.3	6.5	7.0
Health-in-all policies	8.8	6.0	6.1	7.0	6.0	8.1	5.8	6.0
Governance	8.5	6.4	5.9	6.9	8.0	7.2	6.8	7.3
Platforms for interaction	8.3	6.1	5.9	6.9	7.7	5.6	5.6	6.5
Infrastructure support domain median	**8.8**	**6.4**	**6.9**	**7.3**	**8.0**	**6.6**	**6.5**	**7.0**
Overall median	**8.7**	**6.7**	**6.8**		**8.2**	**6.8**	**6.4**	

Note: *I*, importance; *F*, feasibility; *A*, ease of assessment.

All PA-EPI good practice statements were rated by academic and policymaker experts on a 10-point Likert scale for (i) importance (1=relatively unimportant to 10 extremely important), (ii) feasibility (1 = relatively unfeasible to 10 extremely feasible) and (iii) ease of assessment of level of implementation (1 = not at all easy to assess to 10 extremely easy to assess). Median scores per criteria per expert group are shown. Using the expert scores, each statement was ranked by PEN researchers according to the following categories:

(i) Statements rated above the overall median for feasibility, importance and ease of assessment.

(ii) Statements rated above the overall median for feasibility and importance but below the median for ease of assessment.

(iii) Statements rated above the overall median for feasibility and ease of assessment but below the median for importance.

(iv) Statements rated above the overall median for importance and ease of assessment but below the median for feasibility.

(v) Statements rated above the overall median for feasibility but below the median for importance and ease of assessment.

(vi) Statements rated above the overall median for importance but below the median for feasibility and ease of assessment.

(vii) Statements rated above the overall median for ease of assessment but below the median for feasibility and importance.

(viii) Statements rated below the overall median for feasibility, importance and ease of assessment.

**Bold numbers** indicates 'policy', 'infrastructure' and 'overall' median scores for importance, feasibility and assessment by academics and policymakers.

### Step 4: PEN consensus on the PA-EPI framework and monitoring tool

Upon completion of the consultation, a full-day online consensus workshop took place amongst PEN researchers to review academic and policymaker data and reach consensus on the final good practice statements to be included in the PA-EPI. Criteria for good practice statement inclusion involved a review of (i) quantitative data (i.e. median scores obtained from the online consultation, (ii) qualitative data (from the online consultation), (iii) theoretical considerations for tool representativeness of policy and infrastructure domains (a minimum of two good practice statements per domain was agreed) and (iv) pragmatic concerns with regard to the usability of the tool vs. evidence for statement inclusion. The overall conceptualization of the PA-EPI framework remained unchanged from its prototype (figure 1), as well as its two-components ‘Policies’ (with eight domains) and ‘Infrastructure support’ (with seven domains). The consensus workshop led to an agreement on the formulation of the 15 PA-EPI domains’ ideal good practice, and on 45 good practice statements. All were deemed important, feasible and assessable according to the academic experts, policymaker experts and the PEN research team involved in the development process.

## The resulting PA-EPI framework

All PA-EPI domains were deemed relevant to evaluating government progress towards good practice in improving the PA environment and PA behaviour by implementing respective policies and actions. Table 2 presents the final formulation of ideal good practice within the PA-EPI policy domains of education, transport, urban design, healthcare, community-wide programmes, sport and recreation for all, workplaces and public education (including mass media). This provides the context within which to address the inactivity challenge, in part through policy action. The PA-EPI good practice statements, which are the indicators of ideal good practice are also presented in table 2 and provide a monitoring tool for assessment of implementation of policies and actions in which programmes or environmental changes within these settings can be tendered, developed, financed and carried out.^33^ Within the infrastructure support domain, the WHO ‘system building blocks’ are represented.^30^Table 3 outlines ideal good practice and the good practice statements for the PA-EPI infrastructure support domains. These include leadership, governance, monitoring and intelligence, funding and resources, platforms for interaction, developing workforce capacity and health-in-all-policies and are aligned with the Food-EPI, though tailored to PA. Each is a responsibility for governments and statements of good practice call for accountability, transparency, citizen participation, regularity and adequacy in relation to monitoring, resourcing, development and promotion of PA for all.

**Table 2:** PA-EPI policy domains and statements of good practice (*n* = 21)

Domains	Proposed good practice in each domain	Proposed good practice statements
Education (schools)	There are public policies implemented that aim to impact on healthy physical activity environments and promote and support physical activity within the school setting.	E01—evidence informed, quality mandatory physical education that promotes and supports the ideals of equity, diversity and inclusion and adheres to defined standards is part of the curricula in all schools. E02—national and/or sub-national initiatives are in place to promote and support school-related physical activity both at school and in other settings. These initiatives should employ an inter-sectoral approach and collaborative multi-agency partnerships (e.g. links with out-of-school sports clubs, active breaks/recess and walking clubs). E03—there are shared use agreements that utilize school spaces. Community access is supported by initiatives to promote and support opportunities for physical activity for all persons outside of normal school hours. E04—national and/or sub-national policies are in place to promote and support safe active travel to and from school.
Transport	There are public policies to promote and support active mobility for people of all ages and abilities.	T01—regulations are in place that provide a variety of infrastructures to support safe walking and/or cycling and/or wheeling, including measures to calm speed, reduce vehicle traffic and enhance active mobility. T02—there is a funded implementation plan, led by the appropriate level/s of government, to achieve improvements in active travel and increased use of public transport. T03—guidelines and tools to support infrastructure for active mobility and/or transport plans and systems that encourage physical activity are promoted and disseminated.
Urban design	There are public policies enacted at appropriate level/s of government to ensure that evidence-informed urban design principles are implemented to promote and support physical activity and active mobility for people of all ages and abilities.	UD01—policies or regulations that take a ‘health in all’ approach are adopted to reallocate space from motorized transport to active travel and/or recreation purposes. UD02—governments adopt land use policies, and planning processes, consistent with principles of mixed land use, compact urban design and/or provision of green open spaces to support physical activity and reduce motorized transport. UD03—there are guidelines and/or regulations that improve universal and equitable access to safe outdoor and indoor spaces and facilities where people can be physically active.
Healthcare	Public policies implemented within healthcare settings promote and support physical activity, e.g. by providing guidelines and regulations, applying digital health technologies and targeting at-risk groups like older adults.	H01—guidelines and regulations in healthcare include routine screening for physical activity and, for all insufficiently active patients, brief advice and referral to appropriately trained practitioners and/or physical activity opportunities. H03—there are consistent policies for promoting and supporting physical activity in primary and secondary healthcare settings among at-risk groups, such as people with type 2 diabetes and older adults (e.g. protocols for the assessment of the physical activity capacity; accessible, affordable and tailored physical activity programmes; and training for caregivers for delivering physical activity programmes within residential aged care).
Public education/mass media	There are national and/or sub-national public policies implemented to ensure enactment of media/education campaigns that actively promote and support increasing physical activity levels for all ages and abilities.	MM01—there are national and/or sub-national public policies in place that ensure media and education campaigns that promote, and support physical activity are sustained and monitored (e.g. by making them part of, or aligning them with, a national action plan on physical activity and the physical activity guidelines). MM02—there are clear, consistent policies to ensure that multiple media modes/channels (e.g. via posters, social media, radio as well as TV) combined with complementary community initiatives are used to promote the benefits of physical activity and disseminate guidelines, which align with the WHO physical activity recommendations.
Community	There are policies and programmes that promote and support physical activity for all ages and abilities, consistent with relevant recommendations, e.g. by supporting the implementation of whole-of-community events and approaches and promoting the shared use of public spaces and facilities.	C02—public policies are in place to support the implementation of whole-of-community approaches to promote physical activity and networking to strengthen resources and exchange experiences (e.g. WHO Healthy Cities, Active Cities, Partnerships for Healthy Cities). C03—there are public policies in place to foster partnerships for shared use of public spaces and facilities for community-based and community-led physical activity programmes.
Sport & Recreation for All	There are evidence-informed public policies implemented to promote and support sport and recreation for all.	SP01—there are national and/or sub-national evidence-informed ‘Sport and Recreation for All’ policies that prioritize investment in initiatives that target the least active, as well as disadvantaged groups. SP02—there are national and/or sub-national evidence-informed policies or action plans in place that ensure equitable access to sport and recreation spaces and places for all. SP03—there is government support for programmes designed to encourage sports clubs to promote health-enhancing physical activity and other health behaviours (e.g. ‘sports clubs for health’ and ‘health promoting sport clubs’).
Workplace	There are national and/or sub-national policies implemented related to the workplace that promote and support increasing physical activity (e.g. cycle to work initiatives, physically active workplaces) and promote a culture of health for all employees.	W01—there are national and/or sub-national policy initiatives and infrastructure development programmes in place to promote and support safe active travel to and from the workplace. W02—there are concepts and regulations for buildings, plots and the environment in place that promote and support employers to create physically active workplace environments through building design and provision of adequate facilities (both indoor and outdoor).

**Table 3:** PA-EPI infrastructure support domains and statements of good practice (*n* = 24) adapted from the Food-EPI^13^

Domain	Proposed good practice in each domain	Proposed good practice statements
Leadership	The political leadership ensures that there is strong support for the vision, planning, communication, implementation and evaluation of policies to create health-promoting policy environments to improve population physical activity and reduce related inequalities.	L01—there is strong, visible, political support (at the head of state/cabinet level) for creating health-promoting policy environments to improve population levels of physical activity and reduce inactivity related non-communicable diseases and their related inequalities. Political responsibility for health-related physical activity is clearly allocated within the governmental structures. L02—there is a comprehensive up-to-date plan (including timeline, targets, funding, priority policy and programme strategies) linked to national needs and priorities to increase population physical activity. L03—priorities are given to reduce inequalities in relation to inactivity related non-communicable diseases in the comprehensive plan (above). L04—there are clearly defined, evidenced informed population physical activity guidelines for all age groups and for people living with non-communicable diseases, pregnant women and people with disabilities.
Governance	There are government structures in place to ensure transparency and accountability and encourage broad community participation when developing and implementing policies and actions to create healthy physical activity environments and improve population physical activity.	G01—there are reliable procedures to restrict commercial influences related to physical activity environments where there are conflicts of interest with improving population physical activity levels (e.g. restricting lobbying influences that limit physical activity opportunities). G02—there are procedures in place for using evidence in the development of physical activity policies. G03—the government ensures access to and regular dissemination of physical activity guidelines and key documents to the public. G04—the government fosters the cooperation and coordination of all sectors to align with strategic plans to improve the physical activity environment, and where appropriate, promotes civil society participation to develop and implement these plans.
Monitoring and intelligence	There is regular monitoring of population physical activity levels and physical activity environments, systematically linked to the regular monitoring of physical inactivity related non-communicable diseases. Ideally, monitoring should be consistent over time, integrated and occur annually, with more extensive surveys at least every 5 years (e.g. to allow data analysis across all jurisdictions, priority groups). Additionally, policies and major programmes should be evaluated regularly.	MI01—there is regular monitoring of physical activity levels across the life-course based on representative samples, against guidelines/standards/targets. MI02—there is regular monitoring of physical activity environments across all eight policy domains (e.g. walkability and built environment). MI03—physical activity monitoring is systematically linked to the regular monitoring of non-communicable diseases and their related inequalities. MI04—there is regular research and evaluation of policies and major programmes to assess their effectiveness, process and impact on achieving the goals of the physical activity and health plans. MI05—progress towards reducing health inequalities related to social and economic determinants of physical activity is regularly monitored.
Funding and resources	Government funding to support physical activity promotion and research is clearly identified, monitored and sufficient. It is aimed at improving population PA levels, creating active environments, counteracting non-communicable diseases and reducing inequalities.	FR01—the budget spent on physical activity promotion across all policy domains is clearly identified and periodically monitored. FR02—there is a sufficient proportion of total health spending assigned to population physical activity promotion. FR03—a sufficient proportion of total research spending is assigned to population physical activity promotion. FR04—a secure funding stream is available for at least one statutory health promotion agency with an objective to improve population physical activity.
Platforms for interaction	There are coordination platforms and opportunities for synergies across government departments, levels of government and other sectors (e.g. National Government Organizations, private sector and academia) such that policies and actions in physical activity are coherent, efficient and effective in improving environments, population physical activity, reducing inactivity related non-communicable diseases and their related inequities.	PI01—there are robust coordination mechanisms across departments and levels of government to ensure policy coherence, alignment and integration of physical activity, and inactivity related non-communicable disease prevention policies across governments. PI03—there are structures and mechanisms for regular, meaningful and inclusive interactions between government and civil society (academia, professional organizations, public-interest, non-governmental organizations and citizens) on physical activity policies and other strategies to improve population physical activity and health.
Workforce development	Governments have set up systems that provide a platform for population physical activity expertise to ensure that the formulation, implementation and evaluation of physical activity policies and programmes meet population needs.	WD01—to address the challenge of population physical inactivity, there are sufficient resources and people with necessary skills within the government’s workforce (across all eight policy domains). WD02—opportunities for training and professional development are provided to relevant individuals across multiple sectors (e.g. the eight ‘Policy’ domains) regarding the fundamentals of physical activity, its role in public health and effective strategies for physical activity promotion. WD03—support and training systems are in place for relevant professionals (e.g. guidelines, toolkits, training workshops/modules/courses). To ensure uptake, accrediting agencies for professional education, and professional licencing entities should include minimum requirements for initial and continuing education in this domain.
Health-in-All policies	There are processes in place to ensure policy coherence and alignment, and that population health impacts are explicitly considered in the development of all relevant government policies.	HIAP01—there are processes in place to ensure that population physical activity and related health outcomes are explicitly and transparently considered and prioritized in the development of all government policies. HIAP02—there are processes (e.g. health impact assessments) to assess and consider health impacts during the development of policies indirectly related to physical activity.

## Potential process for applying the PA-EPI

**Figure 2:**
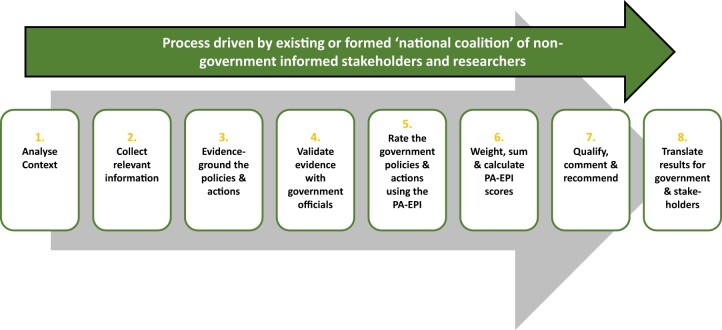
Process for assessing the policies and actions of governments to create healthy physical activity environments and determining the government Healthy PA-EPI (adapted from Swinburn *et al*., 2013)^13^

Emulating the INFORMAS process developed for the Food-EPI (figure 2), the process of conducting the PA-EPI could involve eight steps: (i) analyze context (national or sub-national), (ii) collect relevant information to generate an ‘evidence document’ of implementation of policies and actions by using the PA-EPI good practice statements, (iii) evidence-ground the policies and actions, (iv) validate the evidence with government officials, (v) rate the implementation of policies and actions using the PA-EPI, (vi) weigh, aggregate and calculate the PA-EPI score, (vii) qualify, comment and recommend and (viii) translate the results for government and stakeholders (for more details see: Reference^13^). The purpose of an ‘evidence document’ is to showcase government progress and provide concrete examples as evidence of action or inaction on policy implementation. Conducting the PA-EPI would involve establishing a ‘national coalition’, a group of non-government public health and/or other domains stakeholders to manage the process or, alternatively, select or nominate an existing public health NGO or association to take the lead. This group would rate their government’s recent progress on the creation of a healthy PA environment that is the degree of implementation of policy and infrastructure support in their country against the international best practice for the PA-EPI good practice statements. A 4-point scale for the assessment of level of implementation is used by the Food-EPI.^34^ This scale attributes scores of high, medium, low or none/very little implementation to each good practice statement depending on the quality—strength and comprehensiveness—of the information provided in the evidence document in comparison to international best practice. Ideally, this benchmarking would be a regular, and if possible national event, with scores collated and disseminated publicly. Government ministers and their staff would be sent their scores and rankings, highlighting examples of good progress by their government as well as areas for development to change ill practice to good practice and/or to match or exceed other countries or states. The Active Healthy Kids Global Alliance^3^ and the Global Observatory for Physical Activity^4^ are related initiatives; however, their remit is broader as they monitor global progress in PA surveillance, research over time, with policy indicators as only one of many aspects surveyed. The PA-EPI has a more comprehensive focus on policy. Specifically, it assesses current levels of policy implementation rather than recent progress over time, as the latter may disadvantage governments that already made good progress in the past. However, these initiatives are useful for generating media coverage and responses from bureaucrats and politicians. The Food-EPI has already shown how this process is valuable for stimulating discussion and action nationally.^34^

However, country-specific adaptations might be necessary to account for differences in political culture, to achieve a maximum of stakeholder involvement to build policy capacity, and to ensure high-level political support for an adequate policy response. Initial applications of the PA-EPI should consider these issues, reflect upon and test different options for conducting the PA-EPI and identify their specific strengths and weaknesses. Changing the PA-EPI will compromise comparability and this will also need to be considered. The final step in assessing the level of government policy implementation in the proposed monitoring framework is to combine implementation indicators from all domains across both components into one summary index. Using learnings from INFORMAS and other indexes designed to monitor progress in public health challenges, the relative weighting for each domain and aggregation of individual scores according to defined criteria will assist in this process.^13^

## Future developments and implementation considerations

This article builds on existing work^13^ and, to the authors’ knowledge, is the first attempt at developing a tool that aims to assess the extent of implementation of government policies and actions, with the goal of creating a policy index to assess the healthiness of the PA environment. The conceptual framework and the good practice statements are sufficiently detailed and specific to be used in future PA-EPI rating workshops. However, the final PA-EPI will need to support each good practice statement with specific ‘definitions and scope’ (intended to reduce ambiguity when assessing whether the good practice was implemented successfully), and with examples of international best practice that demonstrate successful implementation of policies that promote PA. These could evolve over time to become benchmarks for monitoring purposes.

Environment policy index development is a dynamic process. As evidence is gathered internationally on the extent of policy implementation by governments at the forefront of the creation of healthy PA environments, concrete examples of best practice are expected to emerge, yielding international benchmark exemplars of best practice. Over time, these benchmarks will strengthen, as governments strive to progressively enhance PA environments, as has been the case for food environments^34^ and tobacco control.^35^

The Food-EPI has shown that the process of conducting an environment policy index is a relatively simple process for small countries (e.g. Ireland) or countries with a not strongly pronounced federalism. It can be more complex for larger countries and countries with federal structures without central authority (e.g. Germany).^13^ Where the responsibility for PA policies is covered by different levels of government, e.g. federal, state and/or local authorities, the implementation of the PA-EPI tool may be more challenging and may require adaptation to cater for the specific needs of the country. In addition, application of the PA-EPI beyond Europe warrants evaluation. The proposed PA-EPI will also need to be tested for functionality, usability, policy relevance, reliability and robustness. The good practice statements and the mechanisms for allocating ratings (as described above), while advised by the Food-EPI process, will require further development in terms of clarity and testing with the proposed national coalitions and government officials. This will also require additional funding and resources to carry out the exercise efficiently and sustainably.

In time, while the primary aim of the PA-EPI is to assess the extent of implementation of government policies and actions to create healthy PA environments, it may also be used for country comparison or benchmarking government policies. A strength of the proposed practice is that, instead of setting a theoretical standard which may never get implemented into practice, a real world, real time comparison can be made by rating the extent of implementation of government action against existing international best practice. This has succeeded in catalyzing action in the food environment, where 12 countries and the European Union have all completed a Food-EPI and have compared best practice between countries. Pilot testing the instrument in high-, medium- and low-income countries will provide insight into the extent that good practice can be made comparable across countries. Like the Food-EPI, the PA-EPI will likely continue to evolve as benchmarks get higher and higher, i.e. a score achieved 1 year may not imply the same level of policy implementation as the same score in the next, because the goalposts will keep moving. Regular updated versions of the good practice statements will be required to keep pace with the changing benchmarks and to improve comparability across countries. Additionally, systems for ensuring quality control and comparability of PA-EPI scores across countries will also need to be considered.

## Conclusions

PA promotion has become an important agenda point for public health agencies. However, the implementation of PA policies poses a few important questions, not least concerning who is responsible for putting policy into practice The PA-EPI framework includes a ‘policy’ and an ‘infrastructure support’ component. Within the policy component there are eight policy domains and within infrastructure support there are seven domains. Each domain has an ‘ideal good practice’, which is underpinned by several good practice statements or indicators. Together they comprise the PA-EPI, a mechanism for monitoring the extent of implementation of government policies and actions to create healthy PA environments. The proposed PA-EPI enables national and international benchmarking and comparisons of public sector policies. It will help identify the major implementation gaps and prioritize actions needed to address critical gaps in government policies and infrastructure support for implementation. This will, in turn, assist in holding governments accountable for their role in the development of a healthy PA environment.

## Supplementary data

Supplementary data are available at *EURPUB* online.

## Supplementary Material

ckac062_Supplementary_Data

## References

[1] World Health Organization. Noncommunicable Diseases. 2020. Available at: https://www.who.int/news-room/fact-sheets/detail/noncommunicable-diseases (14 December 2021, date last accessed).

[2] GBD 2019 Risk Factors Collaborators. Global burden of 87 risk factors in 204 countries and territories, 1990–2019: a systematic analysis for the Global Burden of Disease Study 2019. Lancet 2020;396:1223–49. 33069327 10.1016/S0140-6736(20)30752-2 PMC7566194

[3] Active Healthy Kids Global Alliance. Home. Available at: https://www.activehealthykids.org/ (6 December 2021, date last accessed).

[4] Varela AR , Pratt M, Powell K, et al Worldwide surveillance, policy, and research on physical activity and health: the global observatory for physical activity. J Phys Act Health 2017;14:701–9. 28513338 10.1123/jpah.2016-0626

[5] Whiting S , Mendes R, Morais ST, et al Promoting health-enhancing physical activity in Europe: surveillance, policy development and implementation 2015-2018. Health Policy (New York) 2021;125:1023–30. 10.1016/j.healthpol.2021.05.011 PMC8450183 34120770

[6] World Health Organisation Regional Office for Europe. Physical Activity Factsheets. 2021. Available at: https://www.euro.who.int/en/health-topics/disease-prevention/physical-activity/data-and-statistics/physical-activity-fact-sheets (December 2021, date last accessed).

[7] World Health Organization. Political Declaration of the High-level Meeting of the General Assembly on the Prevention and Control of Non-communicable Diseases. A/RES/66/2 49777. Available at: https://apps.who.int/gb/ebwha/pdf_files/WHA74/A74_10Add1-en.pdf (January 2022, date last accessed).

[8] Guthold R , Stevens GA, Riley LM, Bull FC. Worldwide trends in insufficient physical activity from 2001 to 2016: a pooled analysis of 358 population-based surveys with 1·9 million participants. Lancet Glob Health 2018;6:e1077–86. 30193830 10.1016/S2214-109X(18)30357-7

[9] World Health Organisation. Global Action Plan on Physical Activity 2018-2030: More Active People for a Healthier World. Geneva: World Health Organization, 2018.

[10] Sallis JF , Cervero RB, Ascher W, et al An ecological approach to creating active living communities. Annu Rev Public Health 2006;27:297–322. 16533119 10.1146/annurev.publhealth.27.021405.102100

[11] Swinburn B , Egger G, Raza F. Dissecting obesogenic environments: the development and application of a framework for identifying and prioritizing environmental interventions for obesity. Prev Med 1999;29:563–70. 10600438 10.1006/pmed.1999.0585

[12] Institute of Medicine. Health and Behavior: The Interplay of Biological, Behavioral, and Societal Influences. Washington, DC: The National Academies Press, 2001. 20669491

[13] Swinburn B , Vandevijvere S, Kraak V, et al; INFORMAS. Monitoring and benchmarking government policies and actions to improve the healthiness of food environments: a proposed Government Healthy Food Environment Policy Index. Obes Rev 2013;14:24–37. 24074208 10.1111/obr.12073

[14] Kohl HW , Craig CL, Lambert EV, et al; Lancet Physical Activity Series Working Group. The pandemic of physical inactivity: global action for public health. Lancet 2012;380:294–305. 22818941 10.1016/S0140-6736(12)60898-8

[15] International Society for Physical Activity and Health. ISPAH’s Eight Investments That Work for Physical Activity, Vol. 2021. 2020. Available at: https://www.ispah.org/resources/key-resources/8-investments/ (14 April 2021, date last accessed). 10.1123/jpah.2021-0112 33984836

[16] Lakerveld J , Woods C, Hebestreit A, et al Advancing the evidence base for public policies impacting on dietary behaviour, physical activity and sedentary behaviour in Europe: the Policy Evaluation Network promoting a multidisciplinary approach. Food Policy 2020;96:101873.

[17] Klepac Pogrmilovic B , Ramirez Varela A, Pratt M, et al National physical activity and sedentary behaviour policies in 76 countries: availability, comprehensiveness, implementation, and effectiveness. Int J Behav Nutr Phys Act 2020;17:1–13. 32948193 10.1186/s12966-020-01022-6 PMC7501705

[18] Tcymbal A , Gelius P, Abu-Omar K, et al National focal point network for physical activity promotion – experiences from the European Union (under review). Eur J Public Health. 10.1093/eurpub/ckac079 PMC9421415 36031826

[19] Gelius P , Messing S, Forberger S, et al The added value of using the HEPA PAT for physical activity policy monitoring: a four-country comparison. Health Res Policy Syst 2021;19:1–12. 33588865 10.1186/s12961-021-00681-6 PMC7885477

[20] Noël Racine A , Van Hoye A, Boyd A, et al A scoping review of published research on local government policies promoting health-enhancing physical activity. Int J Sport Policy Polit 2020;12:747–63.

[21] Klepac Pogrmilovic B , O'Sullivan G, Milton K, et al The development of the Comprehensive Analysis of Policy on Physical Activity (CAPPA) framework. Int J Behav Nutr Phys Act 2019;16:60. 31375132 10.1186/s12966-019-0822-5 PMC6679550

[22] Klepac Pogrmilovic B , O’Sullivan G, Milton K, et al A systematic review of instruments for the analysis of national-level physical activity and sedentary behaviour policies. Health Res Policy Syst 2019;17:1–12. 31722717 10.1186/s12961-019-0492-4 PMC6854623

[23] Swinburn B , Sacks G, Vandevijvere S, et al; INFORMAS. INFORMAS (International Network for Food and Obesity/non-communicable diseases Research, Monitoring and Action Support): overview and key principles. Obes Rev 2013;14:1–12. 10.1111/obr.12087 24074206

[24] Horodyska K , Luszczynska A, Hayes CB, et al Implementation conditions for diet and physical activity interventions and policies: an umbrella review. BMC Public Health 2015;15:1250. 26678996 10.1186/s12889-015-2585-5 PMC4683715

[25] Volf K , Kelly L, García Bengoechea E, et al; Policy Evaluation Network (PEN) Consortium. Policy Evaluation Network (PEN): protocol for systematic literature reviews examining the evidence for impact of policies on physical activity across seven different policy domains. HRB Open Res 2021;3:62. 10.12688/hrbopenres.13089.1 PMC8567685 34805740

[26] Woods CB , Volf K, Kelly L, et al The evidence for the impact of policy on physical activity outcomes within the school setting: a systematic review. J Sport Health Sci 2021;10:263–76. 33482424 10.1016/j.jshs.2021.01.006 PMC8167338

[27] Zukowska J , Gobis A, Krajewski P, et al Systematic review of transport policies influencing physical activity. In Abstract book for the ISBNPA 2021 Annual Meeting [online] p. 264. Available at: https://isbnpa.org/news/isbnpa-xchange-2021-abstract-book/ (10 June 2022, date last accessed).

[28] Volf K , Kelly L, Garcia Bengoechea E, Woods CB. Supporting sport for all outcomes through policy action: A systematic review. In Abstract book for the ISBNPA 2021 Annual Meeting [online], 2021, p. 265 Available at: https://isbnpa.org/news/isbnpa-xchange-2021-abstract-book/ (10 June 2022, date last accessed).

[29] Den Braver N , García Bengoechea E, Messing S, et al The impact of mass-media campaigns on physical activity: a review of reviews through a policy lens. Under Rev. 10.1093/eurpub/ckac085 PMC9706123 36444108

[30] World Health Organization. Everybody’s Business: Strengthening Health Systems to Improve Health Outcomes. WHO’s Framework for Action. Geneva, Switzerland: World Health Organization, 2007.

[31] World Health Organization. WHO Director-General Addresses Health Promotion Conference. 2007. Available at: https://www.who.int/director-general/speeches/detail/who-director-general-addresses-health-promotion-conference (6 December 2021, date last accessed).

[32] Sharma G. Pros and cons of different sampling techniques. Int J Appl Res 2017;3:749–52.

[33] Gelius P , Messing S, Goodwin L, et al What are effective policies for promoting physical activity? A systematic review of reviews. Prev Med Rep 2020;18:101095. 32346500 10.1016/j.pmedr.2020.101095 PMC7182760

[34] Djojosoeparto S , Kamphuis CBM, Vandevijvere S, et al; the PEN Consortium, Strength of EU-level food environment policies and priority recommendations to create healthy food environments. *Eur J Public Health* 2022;32:504–11, https://doi.org/10.1093/eurpub/ckac010. 10.1093/eurpub/ckac010 PMC9159309 35265982

[35] World Health Organization. WHO Tobacco Framework Convention on Tobacco Control. Geneva, Switzerland: World Health Organization, 2003.

