# Supplementary material for: The Physical Activity Environment Policy Index for monitoring government policies and actions to improve physical activity
Source: Eur J Public Health. 2022 Nov 29;32(Suppl 4):iv50–8. doi: 10.1093/eurpub/ckac062 (PMC9706113; doi:10.1093/eurpub/ckac062)
Supplement: ckac062_Supplementary_Data [file ckac062_supplementary_data.zip › ckac062_Supplementary_Data/Woods_PA EPI_ SupplMat 1.pdf]

Supplementary Table A. Example of Policy Documents reviewed for development of PA-EPI.

TITLE:	DATE:	ISSUING BODY:	SECTOR:	LINK:
**Global Action Plan for Physical Activity**	2018	WHO	Health	https://www.who.int/ncds/prevention/physical-activity/global-action-plan-2018-2030/en/
**Health economic assessment tool (HEAT) for walking and for cycling**	2017	WHO	Health and Transport	https://www.euro.who.int/__data/assets/pdf_file/0010/352963/Heat.pdf
**Physical activity strategy for the WHO European Region 2016-2025**	2016	WHO Regional office for Europe	Health	http://www.euro.who.int/en/publications/abstracts/physical-activity-strategy-for-the-who-european-region-20162025
**Transforming our world: the 2030 Agenda for Sustainable Development**	2015	UN	Crosscutting	https://sustainabledevelopment.un.org/post2015/transformingourworld
**Global action plan for the prevention and control of noncommunicable diseases 2013–2020**	2013	WHO	Health	https://apps.who.int/iris/bitstream/handle/10665/94384/9789241506236_eng.pdf;jsessionid=F9B05C65144DAC87848E935BE373F813?sequence=1
**Council recommendation on promoting health-enhancing physical activity across sectors**	2013	Council for the European Union	Health	https://eur-lex.europa.eu/legal-content/GA/TXT/?uri=celex%3A32013H1204%2801%29
**Global recommendations on physical activity for health**	2010	WHO	Health	https://www.who.int/dietphysicalactivity/publications/9789241599979/en/
**The Toronto Charter for physical activity: a global call for action**	2010	Physical Activity and Health	Health	http://www.paha.org.uk/Resource/toronto-charter-for-physical-activity-a-global-call-for-action

		Alliance (PAHA)		
**Noncommunicable disease prevention: investments that work for physical activity**	2010	PAHA	Health	http://www.paha.org.uk/Resource/toronto-charter-for-physical-activity-a-global-call-for-action
**EU Physical Activity Guidelines**	2008	European Union	Health	https://ec.europa.eu/assets/eac/sport/library/policy_documents/eu-physical-activity-guidelines-2008_en.pdf
**Steps to health. A European framework to promote physical activity for health**	2007	WHO Regional office for Europe	Health	https://apps.who.int/iris/bitstream/handle/10665/107830/E90191.pdf?sequence=1&isAllowed=y
**The Bangkok Charter for Health Promotion in a Globalized World**	2005	WHO	Health	https://www.who.int/healthpromotion/conferences/6gchp/bangkok_charter/en/
**Linking transport and health in SUMPs; How health supports SUMPs**	2019	European Union	Transport / Health	https://www.eltis.org/sites/default/files/linking_transport_and_health_in_sumps_0.pdf
**EPA Position Paper**	2019	European Parking Association (EPA)	Transport	https://www.europeanparking.eu/media/1583/epa_position-paper.pdf
**MOVING Database**	2020	World Cancer Research Foundation and Co-Create	Cross-cutting	https://www.wcrf.org/wp-content/uploads/2021/12/WCRF-MOVING-Database.pdf
**UNESCO: Quality Physical Education (QPE): guidelines for policy makers**	2015	UNESCO	Education	https://en.unesco.org/inclusivepolicylab/sites/default/files/learning/document/2017/1/231101E.pdf

**MOVING TOWARD ACTIVE TRANSPORTATION: How policies can encourage walking and bicycling**	2016	Active Living Research		https://activelivingresearch.org/sites/activelivingresearch.org/files/ALR_Review_ActiveTransport_January2016.pdf
**Improving Infrastructures for Leisure-Time Physical Activity in the Local Arena**	2011	EU - IMPALA	Urban Design	https://webgate.ec.europa.eu/chafea_pdb/assets/files/pdb/20081208/20081208_oth-02_en_ps_broschure.pdf
**Community Capacity Building for Physical Activity Promotion among Older Adults - A Literature Review**	2017	Publication	Community	Ubert T, Forberger S, Gansefort D, Zeeb H, Brand T. Community Capacity Building for Physical Activity Promotion among Older Adults-A Literature Review. Int J Environ Res Public Health. 2017 Sep 13;14(9):1058. doi: 10.3390/ijerph14091058. PMID: 28902146; PMCID: PMC5615595.
**UNESCO: KAZAN ACTION PLAN**	2017	UNESCO	Education	https://unesdoc.unesco.org/ark:/48223/pf0000252725
**Healthy workplaces: a model for action. For employers, workers, policymakers and practitioners**	2010	WHO	Workplace	https://apps.who.int/iris/bitstream/handle/10665/44307/9789241599313_eng.pdf?sequence=1&isAllowed=y
