# Supplementary material for: The Physical Activity Environment Policy Index for monitoring government policies and actions to improve physical activity
Source: Eur J Public Health. 2022 Nov 29;32(Suppl 4):iv50–8. doi: 10.1093/eurpub/ckac062 (PMC9706113; doi:10.1093/eurpub/ckac062)
Supplement: ckac062_Supplementary_Data [file ckac062_supplementary_data.zip › ckac062_Supplementary_Data/Woods_PA EPI_SupplMat2.pdf]

Supplementary Table B: Comparison Academic and Policymaker data of 'Good Practice Statements' below set criteria following consultation.

**Academic Experts**						**Policymakers**					
**Good Practice Statement**	**I**	**F**	**A**	**Overall Median**	**Rank**	**Good Practice Statement**	**I**	**F**	**A**	**Overall Median**	**Rank**
[b] H03 - There are consistent policies for promoting and supporting physical activity in primary and secondary healthcare settings among at-risk groups, such as people with type 2 diabetes and older adults (e.g., protocols for the assessment of the physical activity capacity; accessible, affordable, and tailored physical activity programmes; and training for caregivers for delivering physical activity programmes within residential aged care).	8.35	6.65	6.57	7.19	1	[b] MM02 – There are clear, consistent policies to ensure that multiple media modes/channels (e.g., via posters, social media, radio as well as TV) combined with complementary community initiatives are used to promote the benefits of physical activity and disseminate guidelines which align with the WHO physical activity recommendations.	7.53	6.78	6.25	6.85	1
[a, b] PI03 – There are structures and mechanisms for regular, meaningful, and inclusive interactions between government and civil society (academia, professional organizations, public-interest, non-governmental organisations, and citizens) on physical activity policies and other strategies to improve population physical activity and health.	8.25	6.32	6.64	7.07	2	[b] H01 – Guidelines and regulations in healthcare include routine screening for physical activity and, for all insufficiently active patients, brief advice, and referral to appropriately trained practitioners and/or physical activity opportunities.	7.95	6.58	6.00	6.84	2
[b] C03 – There are public policies in place to foster partnerships for shared use of public spaces and facilities for community-based and community-led physical activity programmes.	8.19	6.67	6.27	7.04	3	[a, b] W02 – There are concepts and regulations for buildings, plots and the environment in place that promote and support employers to create physically active workplace environments through building design and provision of adequate facilities (both indoor and outdoor).	8.08	6.30	6.03	6.80	3
SP03 – There is government support for 'sports clubs for health' and 'health promoting sports clubs' policies.	7.69	6.65	6.71	7.01	4	UD03 – There are guidelines and/or regulations that improve universal and equitable access to safe outdoor and indoor	7.55	6.65	6.18	6.79	4

						spaces and facilities where people can be physically active.					
[a, b] W02 – There are concepts and regulations for buildings, plots and the environment in place that promote and support employers to create physically active workplace environments through building design and provision of adequate facilities (both indoor and outdoor).	8.08	6.06	6.44	6.86	5	[b] WD02 – Opportunities for training and professional development are provided to relevant individuals across multiple sectors (e.g., the 8 'Policy' domains) regarding the fundamentals of physical activity, its role in public health, and effective strategies for physical activity promotion.	7.90	6.21	6.18	6.76	5
** G05 – There are procedures in place for ensuring transparency in the development of physical activity policies.*	8.18	6.36	5.93	6.82	6	*[*, a] W03 – The government supports companies (both private and public organizations) to promote and support sport participation, activity friendly work practices (e.g., walking phone calls, standing meetings) and physically active social activities in their workplace.*	7.70	6.65	5.90	6.75	6
[, a] W03 – The government supports companies (both private and public organizations) to promote and support sport participation, activity friendly work practices (e.g., walking phone calls, standing meetings) and physically active social activities in their workplace.*	7.67	6.33	6.31	6.77	7	*[*, a] MM03 – There are public policies in place to ensure mass media contain evidence informed focused physical activity messages, appropriate for and tailored to the target audience.*	7.38	6.68	6.13	6.73	7
[b] C02 - Public policies are in place to support the implementation of whole-of-community approaches to promote physical activity and networking to strengthen resources and exchange experiences (e.g., WHO Healthy Cities, Active Cities, Partnerships for Healthy Cities).	7.92	6.29	6.04	6.75	8	[b] HIAP02 - There are processes (e.g., health impact assessments) to assess and consider health impacts during the development of policies indirectly related to physical activity.	7.92	5.95	6.23	6.70	8
** C01 - There are policies consistent with relevant recommendations to promote and support the implementation of free, universally accessible, whole-of-community events as opportunities for being active in local public spaces.*	7.71	6.38	6.04	6.71	9	[a, b] PI03 – There are structures and mechanisms for regular, meaningful, and inclusive interactions between government and civil society (academia, professional organizations, public-interest, non-governmental organisations, and citizens) on	7.72	6.18	6.05	6.65	9

						physical activity policies and other strategies to improve population physical activity and health.					
* SP04 – Inter-sectoral partnerships promoting physical activity through mass participation events are supported by government policy, and/or funding.	7.06	6.67	6.40	6.71	10	MI03 – Physical activity monitoring is systematically linked to the regular monitoring of the prevalence of / risk factors for the main physical inactivity related non-communicable diseases and their related inequalities.	7.74	6.13	6.00	6.62	10
[*, a] _MM03 – There are public policies in place to ensure mass media contain evidence informed focused physical activity messages, appropriate for and tailored to the target audience._	7.54	6.50	6.06	6.70	11	[*, a] _H02 – Policies promote and support the application of digital health technologies that facilitate physical activity promotion in healthcare settings._	6.93	6.60	5.98	6.50	11
[*, a] _PI02 – There are formal platforms (with clearly defined mandates, roles and structures) for regular interactions between national and/or subnational government and physical activity related sectors, and these adopt systematic and transparent accountability processes to identify and ethically manage conflicts of interests._	8.29	5.96	5.79	6.68	12	[a] G01 – There are reliable procedures to restrict commercial influences related to physical activity environments where there are conflicts of interest with improving population physical activity levels (e.g., restricting lobbying influences).	7.97	5.77	5.62	6.45	12
[*, a] _H02 – Policies promote and support the application of digital health technologies that facilitate physical activity promotion in healthcare settings._	6.71	6.31	6.63	6.55	13	[a] WD01 – The capacity (numbers and skills) of the government's public health workforce is aligned with the scale of the physical inactivity problems of the population and the amount of government resources for health.	7.74	5.67	5.62	6.34	13
[a] G01 – There are reliable procedures to restrict commercial influences related to physical activity environments where there are conflicts of interest with improving population physical activity levels (e.g., restricting lobbying influences).	8.50	5.43	5.46	6.46	14	[*, a] _PI02 – There are formal platforms (with clearly defined mandates, roles and structures) for regular interactions between national and/or subnational government and physical activity related sectors, and these adopt systematic and transparent accountability processes to identify and ethically manage conflicts of interests._	7.69	5.54	5.54	6.26	14

[a] WD01 - The capacity (numbers and skills) of the government's public health workforce is aligned with the scale of the physical inactivity problems of the population and the amount of government resources for health.	8.07	5.32	5.86	6.42	15						

Key:
I = Importance; F = Feasibility; A = Ease of Assessment; Rank = 'Good Practice Statements' below set criteria following consultation were ranked from highest to lowest.
* denotes 'Good Practice Statements' that were removed from PA-EPI following expert/policymaker consultation and WP1 partner consensus workshop (n=7; H02, MM03, C01, SP04, W03, G05, PI02).
[a] = 'Good Practice Statements' considered for removal that were common to both Academic Expert and Policymaker Consultations (n=8)
[b] = 'Good Practice Statements' that were exempt from consideration for removal to maintain the criterion of having a minimum of two 'statements' within each domain (n=9)
