# Supplementary material for: The Physical Activity Environment Policy Index for monitoring government policies and actions to improve physical activity
Source: Eur J Public Health. 2022 Nov 29;32(Suppl 4):iv50–8. doi: 10.1093/eurpub/ckac062 (PMC9706113; doi:10.1093/eurpub/ckac062)
Supplement: ckac062_Supplementary_Data [file ckac062_supplementary_data.zip › ckac062_Supplementary_Data/Woods_PA EPI_SupplMat3.pdf]

Supplementary Table C: List of academic experts who completed the PA-EPI online consultation and consented to acknowledgement.

Name	Country	Representative Of
Dr. Krelmin Wickramasinghe	Russia	NCD, WHO
Dr. Bojana Klepac Pogrmilovic	Australia	Institute for Health & Sport, Victoria University
Dr. Sonja Kahlmeier	Switzerland	Department of Health, Swiss Distance University of Applied Science FFHS
Prof. John Spence	Canada	University of Alberta
Prof. Michael Pratt	USA	Institute for Public Health, University of California
Dr. Tracy Nau	Australia	Sydney School of Public Health
Dr. Catherine Carty	Ireland	UNESCO Inclusive Policy Lab / Institute of Technology Tralee
Prof. Anne Vuillemin	France	Université de la Cote d´Azur
Prof. Charlie Foster	UK	University of Bristol
Prof. Andy Pringle	UK	University of Derby
Prof. Jo Salmon	Australia	Deakin University
Prof. Greet Cardon	Belgium	Ghent University
Prof. Nanette Mutrie	Scotland	University of Edinburgh
Dr. Andrew Milatt	Australia	NSW Public Health
Kate Oldridge-Turner Margarita Kokkorou	UK	Head of Policy & Public Affairs, CO-CREATE, World Cancer Research Fund International
Prof Palma Chillón	Spain	University of Granada
Arthur Furtado	Luxembourg	European Commission, DG Santé
Prof. Klaus Pfeifer	Germany	FAU Erlangen-Nürnberg
Prof. Kevin Patrick	USA	University of California San Diego
Prof. Kevin Leydon	Ireland	NUI Galway
Prof. Jim Sallis	USA	San Diego
Prof. Agnieszka Jaszczak	Poland	UWM Olsztyn
Prof. Karsten Zimmermann	Germany	TU Dortmund
Prof. Sabine Baumgart	Germany	TU Dortmund
Prof Melody Smith	New Zealand	University of Auckland
Prof. John Oetzel	New Zealand	University of Waikato
Prof. Melinda Craike	Australia	Mitchell Institute for Health Policy, Victoria University
Prof. Jasper Schipperijn	Denmark	University of Southern Denmark
Prof. Lucie Levesque	Canada	Queen's University, Canada
Prof. Niamh Murphy	Ireland	Waterford Institute of Technology
Prof. Rochelle Eime	Australia	Victoria University
Prof. Winfried Banzer	Germany	Goethe-Universität Frankfurt
Dr. Richard Bailey	UK	International Council of Sport Science and Physical Education
Benny Cullen	Ireland	Sport Ireland
Dr. Aurelie van Hoye	France	University of Lorraine
Dr. Sami Kokko	Finland	University of Jyväskylä
Dr. Lindsey Reece	Australia	University of Sydney
Dr. Alejandra Jauregi	Mexico	National Institute of Public Health
Apl-Prof. Christine Joisten	Germany	DSHS Köln
Dr. Anne-Maree Parrish	Australia	Faculty of Social Sciences, University of Wollongong, Australia
Dr. Josephine Chau	Australia	Macquarie University
Prof. Leen Haerens	Belgium	University of Ghent
Prof. Hal Lawson	USA	University of Albany
Prof. Ann McPhail	Ireland	University of Limerick

Dr. Jenna Lorusso	Ireland	University of Limerick
Dr. Paul Kelly	Scotland	University of Edinburgh
Prof. Carole Clavier	Canada	UQAM Montréal
Prof. Tanya Berry	Canada	University of Alberta
Radoslaw Czapski	Poland	World Bank, Poland
Dr. Lech Michalski	Poland	Pomeranian Regional Planning Office
Prof. Jenny Mindell	UK	University College London
Dr. David Ogilvie	UK	University of Cambridge
Prof. Ruth Hunter	UK	Queens University Belfast
Dr. Sandy Mandic	New Zealand	Auckland University of Technology
Dr. Lorraine Darcy	Ireland	Technological University Dublin
Prof. William Bellew	Australia	University of Sydney
Dr. Elizabeth Ablah	USA	University of Kansas
Dr. Aleksandra Romanowska	Poland	Gdansk University of Technology
Dr. Antje Hebestreit	Germany	Leibniz Institute for Prevention Research and Epidemiology – BIPS, Bremen
Dr. Carlijn Kamphuis	The Netherlands	Faculty of Social and Behavioural Sciences, Utrecht University
Dr. Celine Murrin	Ireland	School of Public Health, Physiotherapy, and Sports Science, University College Dublin
Dr. Janas Harrington	Ireland	School of Public Health, University College Cork
Prof. Knut Inge Klepp	Norway	Norwegian Institute of Public Health, Oslo
Dr. Maartje Poelman	The Netherlands	Wageningen University & Research, Wageningen
Dr. Peter von Philipsborn	Germany	Pettenkofer School of Public Health, Ludwig-Maximilians-University Munich
Dr. Piotr Romaniuk	Poland	Medical University of Silesia in Katowice
Sanne Djojosoeparto	The Netherlands	Utrecht University, Utrecht

*Note. An additional five individuals took part but asked to remain anonymous.
